# A Clinical and Neuropathological Study of Chinese Patients with Diabetic Peripheral Neuropathy

**DOI:** 10.1371/journal.pone.0091772

**Published:** 2014-03-11

**Authors:** Guangren Li, Chenglin Sun, Yanjun Wang, Yujia Liu, Xiaokun Gang, Ying Gao, Fei Li, Xianchao Xiao, Guixia Wang

**Affiliations:** 1 Department of Endocrinology and Metabolism, The First Hospital of University, Jilin University, Changchun, Jilin, China; 2 Department of Neurology, China-Japan Union Hospital, Jilin University, Changchun, Jilin, China; 3 Department of Endocrinology and Metabolism, The Second Hospital of University, Jilin University, Changchun, Jilin, China; University of Florida, United States of America

## Abstract

**Objective:**

To examine whether the neuropathological and metabolic changes of peripheral nerves are correlated to clinical features in diabetes mellitus type 2 patients with peripheral neuropathy.

**Methods:**

147 type 2 diabetic patients with signs/symptoms of diabetic peripheralneuropathy (DPN) aged 53.4±12.3 years and 134 healthy volunteers aged 55.5±11.7 years were investigated for fasting plasma glucose (FPG), hemoglobin A1C (HbA1c), and red blood cell sorbitol (RBC sorbitol) in addition to nerve conduction velocity (NCV). Among the 147 diabetic patients, 10 patients underwent superficial peroneal nerve biopsy for light and electron microscopy.

**Results:**

In the experimental group, the levels of HbA1c and RBC sorbitol showed significant increase compared with the controlled group, whereas motor nerve conduction velocity (MNCV) and sensory nerve conduction velocity (SNCV) both showed decline and SNCV decreased at a greater extent. Morphologically, there were various degrees of nerve fiber loss, associated with axon degeneration and capillary luminal narrowing in 10 patients undergone nerve biopsy.

**Conclusion:**

The metabolic change of sorbitol, the consequently observed changes in NCV and histopathology of peripheral nerves are positively correlated with the duration of diabetes and overall level of blood glucose.

## Introduction

Diabetic peripheral neuropathy (DPN) is a common long-term complication of diabetes mellitus, which can affects at least 50% of patients with type 1 and type 2 diabetes and is a leading cause of foot amputation [1]. It may affect sensation, movement, and gland or organ function depending on the type of nerves involved [2]. Sensory neuropathy may cause numbness to touch and vibration, reduced position sense causing poorer coordination and balance, and reduced sensitivity to temperature change and pain, spontaneous tingling, burning pain, and skin allodynia; motor neuropathy may cause impaired balance and coordination and, most commonly, muscle weakness [3], [4]. The pathogenetic mechanisms of DPN have not been well understood, but metabolic and vascular deficiencies caused by diabetes may be important contributors [5]. Previous studies have shown that increased aldose reductase activity, advanced glycation/glycoxidation, oxidative-nitrative stress, activation of protein kinase C, poly (ADP-ribose) polymerase, and impaired neurotrophic support are involved in the pathogenesis of DPN [6], [7], [8], [9]. In this paper, we tried to find out whether the duration of diabetes and the long term unsatisfied blood glucose control have relationship with the metabolic and neuropathological changes in DPN patients.

In this study, we studied the clinical presentation, serum markers for disease progression, NCV and morphology of peripheral nerves in DPN patients.

## Patients and Methods

### Patients

The control group was consisted of 134 healthy volunteers. The experimental group included 147 type 2 diabetes patients with diabetic peripheral neuropathy, and 10 patients (Table 1, 2)received the morphological study of peripheral nerves.The study was approved by the China Research Ethic Committee, and all participants have provided their writteninformed consent before participating in this study.

**Table 1:** Clinical data of individual patients.

Patients	Sex	Age (years)	Clinical Manifestation	Neurological dysfunction		
				Sensory	Motor	Autonomic
1	M	45	Hand and foot numbness and weakness, poor fine finger movement, muscle atrophy, urinary retention, reflex decline in knee and ankle	+++	++	+
2	F	38	Hand and foot numbness and pain, diarrhea, tactile decline in knee and ankle, reflex decline in ankle	++	-	+
3	F	56	Hand and foot pain, urinary retention, tactile decline in wrist and ankle, reflex decline in ankle	++	-	+
4	M	52	Hand and foot numbness and weakness, muscle atrophy in fingers, squating and standing difficulties, diarrhea, tactile loss of tuning fork, reflex decline in knee and ankle	+++	+++	+
5	M	58	Hand and foot numbness, hypertension, black ulceration in the left great toe, pulsation decline in the dorsalis pedis artery	+	-	-
6	M	46	Feet numbness, sweating, urinary retention, reflex decline in knees and ankles	++	-	++
7	M	51	Right foot numbness, vision loss, intermittent diarrhea, reflex decline in knees and ankles	+	_	+
8	F	43	Feet numbness, fast resting heart rate, shortened PR, reflex decline in knees and ankles	+	-	+++
9	M	41	Intermittent numbness	+	-	-
10	F	46	Intermittent foot numbness and pain	+	-	-

+ ∼ +++ represents the various extents of neurological dysfunction based on the symptoms and physical examination.

**Table 2:** Duration and pathologic data of individual patients.

Patients	Duration (years)		Perineurium Edema	Myelinated Fiber Decrease	Vascular injury
	DM	DPN			
1	17	6	++	+++	++
2	8	2	+	++	+
3	6	1	+	+	+
4	12	5	++	+++	++
5	6	2	++	+	+++
6	5	1	+	+	+
7	8	2	+	++	+
8	7	2	+	+	+
9	3	0.25	+	-	-
10	3	0.17	+	-	-

+ ∼ +++ represents the various extents of neurological and vascular changes

based on the morphological study of peripheral nerves.

### Determination of blood samples

Blood samples were drawn from fasting patients. FPG level was determined using One Touch blood glucometer (Johnson&Johnson Company, USA). HbA1C was determined using DCA Vantage-HbA1c analyzer (Siemens, Eschborn, Germany). Red blood cell sorbitol was measured by using the sorbital dehydrogenase ELISA kit (Megazyme International Ireland Ltd., Wicklow, Ireland).

### Assessment of nerve conduction velocity (NCV)

NCV was evaluated by using a DantecKeypoint electromyogram system (Dantec Medical, Skovlunde, Denmark). Testing was standardized for temperature, side of testing, stimulation protocol, averaging of sensory potentials andmeasurement of latencies and amplitudes.The velocity of motor and sensory nerve conductions were measured using surface electrodes in the median and common peroneal nerves from both arms and legs.

### Morphological study of peripheral nerves

A 1-cm long incision was made behind the left lateral malleolus. The skin was retracted, and a branch of the superficial peroneal nerve was gently separated from adjacent structures by a blunt instrument. A 1–1.5 cm segment of the nerve was obtained and divided into two portions. The first portion wasfixed in formalin at 4°C and embedded in paraffin. The fixed tissue was sectioned at 5 μm with a rotary microtome. The sections were deparaffinized and dehydrated in xylene and then a descending ethanol series. The sections were stained by H&E and examined by light microscopy. The second portion was fixed in a mixture of 4% glutaraldehyde and 1% acid phosphate buffer. Following alcohol dehydration, the samples were embedded in Epon 812. Transverse sections (0.5–1 μm thick) were stained with Toluidine Blue and examined by light microscopy. Ultra-thin sections were stained with uranium acetate and lead citrate and examined by electron microscopy.

### Statistical analysis

All data was presented as mean±SD, and data of each measurement of the two groups were analyzed using t-test.

## Results

**Figure 1:**
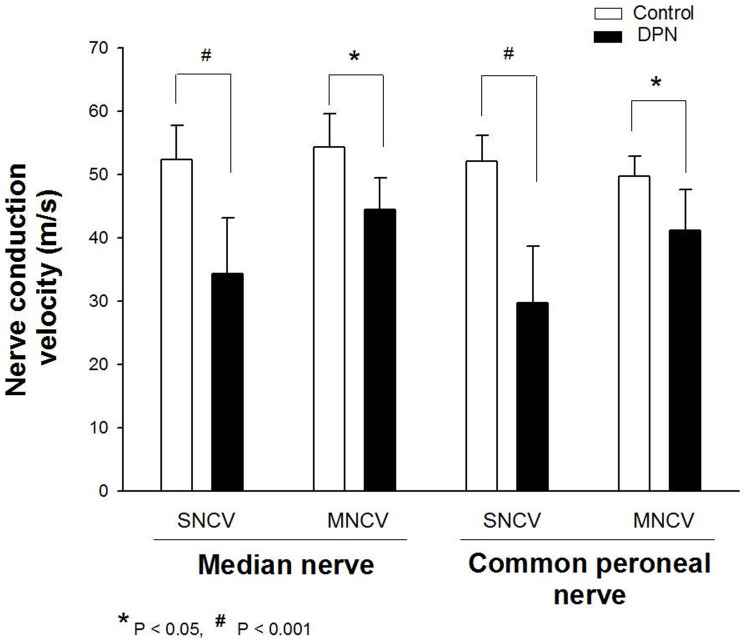
Assessment of nerve conduction velocity (NCV) was performed in controlled group and experimental group. The SNCV and MNCV of both the median and common peroneal nerves have shown a statistical significant reduction in DPN groupcompared with controlled group.

**Figure 2:**
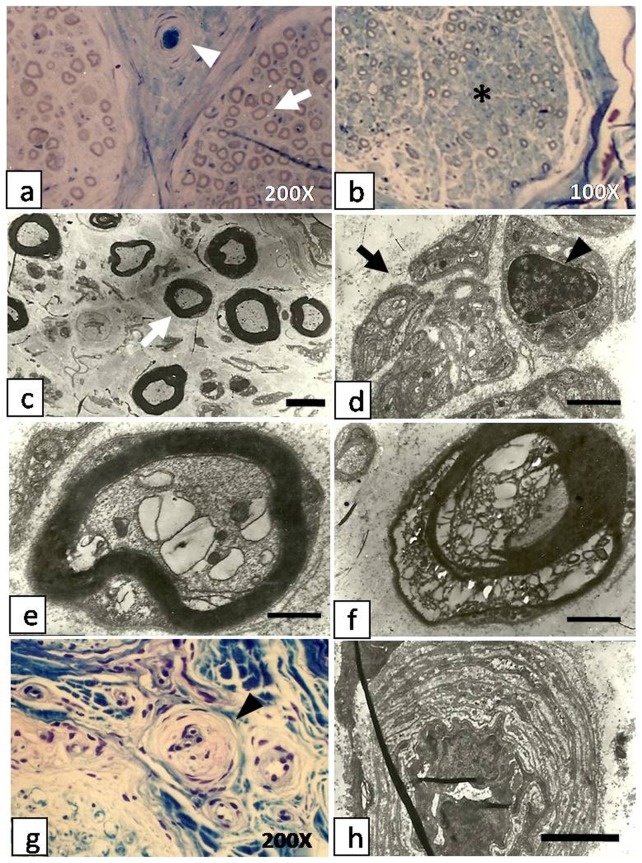
Cross section biopsies of sural nerves in patients with diabetic peripheral neuropathy. a. Semi-thin section of sural nerve of case5 shows mild reduction in the number of nerve fibers (white arrow), and an arteriole plugged by a thrombus(white arrowhead). b. Semi-thin sections of sural nerve from case 1 shows severe loss of myelinated axons and increased endoneurial collagen (asterisk). C. and d. Semi-thin sections showdegeneration and regeneration of myelinated (white arrow) and unmyelinated (black arrow) fibers and proliferation of Schwann cells (black arrowhead) (case 3 and 6). e. and f. Semi-thin sections of a single myelinated axon showdegenerating myelin sheaths, absence of lamellar structures and balloon cell degeneration of the neuronal body (e: case 3); the axon shows almost complete structural disintegration (f: case 1). g. Section stained by Toluidine blue and examined by light microscopy shows luminal narrowing of epineural arterioles (black arrowhead). h. Electron micrograph of perineural arteriole shows luminal narrowing, endothelial thickening, basement membrane thickening and vascular wall thickening. Scale bar for (c–h): 2.5 μm, (d–f): 500 nm.

Clinical profiles of the diabetic subjects and nerve conduction velocity are shown in Table 3. Compared with the controlled group, the DPN group showed increased levels of FPG, HbA1C, RBC sorbitol, action potential duration and the incubation period, while decreased NCV. In the DPN group, HbA1C and RBC sorbitol have shown a statistical significant increase, while SNCV and MNCV in both the median and common peroneal nerves have shown a statistical significant reduction compared with controlled group (Fig. 1). Specifically, there were 10 individual patients who received nerve biopsies. Patients 1 and 4 with a long history of diabetesshowed muscle weakness and atrophy as well as poor fine motor movement of the fingers, and they had the most severe histopathological alternations among the ten patients. In cases 9 and 10 where the two patients with a relatively short history of diabetes, the histopathological alternations were not obvious.The other 6 patients showed varying degrees of alteration (Fig. 2).

**Table 3:** Clinical Profiles, Results of Lab study and NCV (Data presented as mean±SD).

Characterististics	Control (n = 134)	DPN (n = 147)
Sex (male/female)	75/59	89/58
Age (years)	53.4±12.3	55.5±11.7
Duration of diabetes (years)	N/A	7.3±4.3
Duration of DPN (years)	N/A	2.6±1.8
FPG (mmol/L)	5.28±1.41	11.54±1.67
HbA1C (%)	5.23±1.02	8.27±1.24 ^*^
RBC Sorbitol (nmol/g Hb)	44.02±7.83	65.64±11.59 ^#^
Action potential duration (ms)		
Median nerve	9.05±1.62	11.62±2.45
Common peroneal nerve	9.15±1.67	11.44±2.34
Incubation period (ms)		
Median nerve	3.67±0.72	5.08±0.89
Common peroneal nerve	4.32±0.77	6.85±2.06
Median Nerve (m/s)		
SNCV	52.37±5.42	34.28±8.83 ^#^
MNCV	54.28±5.32	44.40±5.07 ^*^
Common Peroneal Nerve (m/s)		
SNCV	52.07±4.12	29.70±8.88 ^#^
MNCV	49.77±3.14	41.14±6.42 ^*^

All values are mean ±SD. * P<0.05; # P<0.001.

Edema of the perineurium was observed by light microscopy in all patients. There was significant decrease of myelinated fibers in cases 1 and 8 (Fig. 2b). For those with advanced axonal neuropathy (cases 1 and 4), there was loss of myelinated axons and increased endoneurial collagen. Examination with electron microscope showed expansion and vacuolization of the microtubules along with mitochondrial swelling (Fig. 2e, f). There was also degeneration of the myelin sheaths shown by crumbled and/or absence of lamellar structures. These findings were more prominent in cases 1 and 4. The degeneration of nerve fibers was accompanied by regeneration of myelinated and unmyelinated fibers and proliferation of Schwann cells (Fig. 2c, d).

Vascular changes with significant reduction in the number of perineural arterioles were found in all cases. The remaining arterioles showed proliferation and hypertrophy of endothelial cells, thickening of vascular walls and narrowing of the luminal cavity (Fig. 2g). These changes were more prominent in case 5 where an obliterating thrombus was also noted (Fig. 2a). Similar findings in addition to noticeable thickening of the basement membranes were seen by electron microscopy (Fig. 2h).

## Discussion

Peripheral neuropathy is a common complication of diabetes where symptoms related to sensory and autonomic nerves damage appear earlier than those related to motor nerves damage. Sensory and autonomic nerves, being thinner and longer, may be more vulnerable to metabolic alterations than motor nerves[10], [11]. In cases 1 and 4, the decreased nervebundles,the significant reduction of myelinated fibers and serious destruction of the myelin sheaths were demonstrated by pathology studies, but in cases 9 and 10, endoneurium edema wasthe only morphological finding. Intravascular thrombosis was found in case 5, who had the most severe vascular injury according to physical examination as it has been demonstrated in Table 2. Considering that the case 1 and 4 have the longest diabetes duration along with the most severe neurological dysfunction, while case 9 and 10 are just the opposite, we tentatively put forward that the histopathological changes of peripheral nerves may due to the duration of diabetes since the ten cases were randomly selected from the 147 DPN patients.

The FPG and HbA1ccan be used as standards to demonstrate the short and long term blood glucose control respectively. Although the levels of serum blood glucose, HbA1c and RBC sorbitol were significantly increased in all DPN patients, the manifestations of the disease were different among individual patients with various severity and duration of diabetes. The level of HbA1c, but not that ofserum glucose, correlated with the development and severity of neuropathy. This indicatesthat severe diabetic neuropathy may be related to the duration of diabetes and poor long-term blood glucose control. Under normal physiological conditions, metabolism of glucose proceeds through its phosphorylation by hexokinase/glucokinase, with about 3% of the glucose being metabolized in the polyol pathway; however, under conditions of elevated glucose levels, there is a saturation in the glycolytic pathway at the hexokinase step,which causes the excess glucose to be diverted to the polyol pathway where it is further metabolized to sorbitol and then to fructose due to activation of aldose reductase and sorbitol dehydrogenase (SDH) [12], [13]. Since sorbitol cannot cross cell membranes, when it accumulates, it produces osmotic stress inside cells by drawing water into the insulin-independent tissues [14], [15], [16].

Based on the patient's history, the physical examination and laboratory investigation, we believe that the severity of diabetic neuropathy isclosely correlated to the durationof diabetes, overall level of blood glucose and metabolic change of sorbitol; and the nerve conduction velocity and morphologic changes of peripheral nerves reflectthe degree of diabetic neuropathy. In cases 9 and 10, the SNCV were slightly decreased and neuropathy was only accompanied by the endoneurial edema; similar results were reported by Yagihashi S.They demonstrated that the endoneurium edema was generated duringthe early stage of diabetic neuropathy and was related with activation of the polyol pathway [17]. Many studies reported that the blood sorbitol levels were positively correlated with that in lens and sciatic nerve [14], [18], [19]. In this study, RBC sorbitol of DPN patients were increased, which indirectly indicated that the nerve sorbitollevel increased and thusled to osmotic imbalance of neuron cells and further affected the metabolism of inositol and Na^+^/K^+^-ATP enzyme [20]. In addition, some studies have shown that the polyol pathway is the major source of diabetes-induced oxidative stress in nerves [21]. Imbalance between intracellular and extracellular Na^+^/K^+^ is possible to causeendoneurium edema, which will further evoke the demyelination and degeneration of nerves that can aggravate the nerve conduction block, especially in severe cases such as 1 and 4.

In case 1 and 8, the microangiopathy was demonstrated by neuropathological study, which showed not only reduction in the number of capillariesamong the nerve fibers but also the endothelial cells proliferation and luminal stenosis, which were bound to reduce nerve blood flow and oxygen levels in the endoneurium and further induced nerve degeneration and necrosis because of its inability to keep up with the metabolic and nutritional demands of the axons. The decreased or unaltered vascular density in DPN was reported by Younger DS [7] and Said G [22] and similar results were found in our study. We have demonstrated that vascular density was unaltered in most of our patients with DPN, but decreased capillaries and vascular thrombosis were found in case 5 who presented with diabetic foot.

In conclusion, the metabolic change of sorbitol, the consequently observed changes in NCV and histopathology of peripheral nerves are positively correlated with the duration of diabetes and overall level of blood glucose. We have demonstrated that severity of diabetic peripheral neuropathy is closely correlated with the duration of diabetes and the long term unsatisfied blood glucose control. A combination of metabolic and vascular defects has been implicated in the pathogenesis of diabetic neuropathy.It is concluded that robust inhibition of metabolic flux through the polyol pathway in peripheral nerve, and decreased oxidative stress will likely result in substantial clinical benefit when treating and preventing the currently intractable condition of diabetic peripheral neuropathy. The potential factors contributing to nerve ischemia include structural defects in the endoneurial microvasculature along with rheological abnormalities, and abnormalities in vasoactive agents that regulate nerve blood flow; thus, vasodilators could potentially prevent or ameliorate nerve dysfunctions. However, expanded data base and clinical cases can provide more information for histopathological study and contribute to a more confirmative and conclusive further research, and this is what we would continue to work on.
